# Heat penetration and thermocouple location in home canning

**DOI:** 10.1002/fsn3.185

**Published:** 2014-12-09

**Authors:** Mark R Etzel, Paola Willmore, Barbara H Ingham

**Affiliations:** Department of Food Science, University of Wisconsin-Madison1605 Linden Drive, Madison, Wisconsin, 53706

**Keywords:** Biot number, canning, cold spot, consumer, heat penetration, heat transfer, thermal processing

## Abstract

We processed applesauce, tomato juice, and cranberries in pint jars in a boiling water canner to test thermal processing theories against home canning of high-acid foods. For each product, thermocouples were placed at various heights in the jar. Values for *f*_h_ (heating), *f*_cl_ (cooling), and *F*_82.2°C_ (lethality) were determined for each thermocouple location, and did not depend substantially on thermocouple location in accordance with heat transfer theory. There was a cold spot in the jar, but the cold spot during heating became the hot spot during cooling. During heating, the geometric center was the last to heat, and remained coldest the longest, but during coooling, it was also the last to cool, and remained hottest the longest. The net effect was that calculated lethality in home canning was not affected by thermocouple location. Most of the lethality during home canning occurred during air cooling, making cooling of home canned foods of great importance. Calculated lethality was far greater than the required 5-log reduction of spores in tomato juice and vegetative cells in cranberries, suggesting a wide margin of safety for approved home-canning processes for high-acid foods.

## Introduction

Evidence indicates a revival of interest in home canning (Moskin 2009; Dickerson 2010). However, studies by the National Center for Home Food Preservation (Andress et al. 2002; D'Sa et al. 2007) suggest that consumers engaged in home canning may not be following up-to-date, tested recipes, potentially putting the health of these consumers and their families at risk.

In order to establish safe home-canning methods, it is necessary to verify that processes deliver sufficient thermal lethality. Thermal processing operations in the canning industry aim to ensure adequate destruction of expected spoilage organisms and pathogens in the product based on reliable microbial thermal-death-time information. The primary public health concern associated with low-acid canned food is the formation of botulinal toxin in the container (Weddig 2007); with acid or acidified canned foods the threat to public health is from *Escherichia coli* O157:H7 (Breidt et al. 2010) or *Listeria monocytogenes* (Breidt et al. 2014).

The design of adequate thermal processes requires an understanding of how a product heats and cools under processing conditions. Bee and Park (1978) noted that the design of heat processes is based on the measurement of temperatures in the coldest portion of the container at various time intervals. Weddig (2007) stated that the slowest heating region, or cold spot, of the container depends on product type, container type and size, processing method, and the heat transfer mechanism. Typical heat transfer mechanisms in canned foods are convection, conduction, and a combination of convection and conduction. The assumed cold spot of a can or jar heated in a stationary vessel is one-third from the base for convection heating products, and at the geometric center of the container for conduction heating products heated by conduction (Potter and Hotchkiss 1998).

In order to test these thermal processing theories as they apply to home-canning operations, we processed applesauce, tomato juice, and cranberries in heavy syrup in pint-size jars in a boiling water canner (BWC) following current research-based methods (USDA 2009). Our goals were to study the thermal adequacy of home-canning processes for typical high-acid foods, to establish the contribution of various portions of the canning process to overall lethality, and to determine the impact of thermocouple placement on calculated total process lethality.

## Materials and Methods

### Equipment

A 23-liter BWC (Victorio VKP1055; Victorio Kitchen Products, Orem, UT) was used in this study and operated according to standard home-canning recommendations (USDA 2009). Temperature of the canner water was measured with a type-T thermocouple with stainless steel tubing (Ecklund-Harrison Technologies Inc., Ft. Myers, FL) placed at a depth of 15 cm in the water column. Glass canning jars (16 fl oz, 473 mL; PT) with standard 2-piece lids (eight jars per trial) were equipped with adjustable needle type-T thermocouples placed at various depths within the eight jars, beginning at the geometric center (7.5 cm above the base) and descending in roughly 1-cm increments to a depth of 1.5 cm above the base. Thermocouples were held in place by stuffing boxes attached to a datalogger (TechniCAL, New Orleans, LA) equipped with Calsoft 32 software (TechniCAL). Temperature was recorded at 1 min intervals for each thermocouple.

### Food processing

Experiments were conducted with laboratory-prepared tomato juice, cranberries in heavy syrup, and applesauce, following current, approved home-canning recipes (USDA 2009). The BWC was filled with 10 L of tap water (24°C) prior to each run. Freshly prepared tomato juice, cranberries in heavy syrup (40% w/v sucrose), or applesauce were prepared as directed, heated to 82°C and filled into hot (82°C) PT-jars. Headspace was adjusted to ½-inch (1.27 cm) and the weight of each jar was recorded. Target fill weight was 450, 460, and 450 g, for tomato juice, applesauce, and cranberries in syrup, respectively. Fill weight of cranberries was achieved with a target weight of cranberries (220 g) and syrup (230 g). Canning lids fitted with thermocouples were placed on jars and closed until fingertip tight. Prepared jars were placed in a BWC with water preheated to 82±1°C. Processing time started when the thermocouple in the water registered 99±1°C. Jars were processed for 35, 15, or 15 min, for tomato juice, cranberries, or applesauce, respectively. At the end of the processing time, jars were removed from the canner and placed on a laboratory bench to cool to ∼54°C. Three separate trials were conducted for each food product.

### Data handling

Time/temperature data were plotted for each thermocouple location for each product and each run. The Ball equation (eq. ) (Singh and Heldman 2009, pp. 358–365) was used to calculate *f*_h_ and *j*_h_, and *f*_cl_ and *j*_cl_, for the heating and cooling potions of the curve, respectively, at each thermocouple placement. 

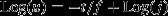
1where *u* = (*T* − *T*_*a*_)/(*T*_0_ − *T*_*a*_) and *T* = thermocouple temperature (°C); *T*_*a*_ = heating medium temperature (°C); *T*_0_ = thermocouple temperature at the start of the heating or cooling process; *f* = the time for a one-log reduction in *u* (*f* = −1/slope of the linear portion of the semilogarithmic plot, that is, where *u* < 0.7); and *j* = lag factor for heat penetration to the point of temperature measurement. The lag factor (*j*) does not depend on the kind of material being heated, only on the object shape, thermocouple location, and initial temperature distribution. The heat penetration rate (*f*) does not depend on the thermocouple location, only on the kind of material being heated and the object shape.

Integrated process lethality was determined for the heating (*F*_h_) and cooling (*F*_c_) portions of the curve and for the overall curve (*F*) by finding the area under the curve according to equation 2 (Stoforos 2010), assuming *E. coli* O157:H7 as the target pathogen. 

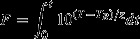
2where *T* = product temperature; *T*_R_ = reference temperature; *t* = time; *z* = temperature increase required for a 10-fold decrease in the decimal reduction time (*D*_T_). For *E. coli* O157:H7, *z* = 5.56°C and *D*_T_ = 0.00008 min at 82.2°C (Mazzotta 2001).

Results for *f*_h_, *f*_cl_, and *F*_82.2°C_ (heating, cooling, total) were compared using analysis of variance (SAS version 9:2, SAS Institute Inc., Cary, NC) with significance level set to *P* = 0.05. The experiment was a randomized complete block design with three trials per food product and eight thermocouple locations per trial. Data were analyzed by trial and by thermocouple. In variables that had a significant difference between thermocouples within each trial, a protected least significant difference (LSD) test was used to pinpoint the exact differences in the experiment.

## Results and Discussion

**Figure 1:**
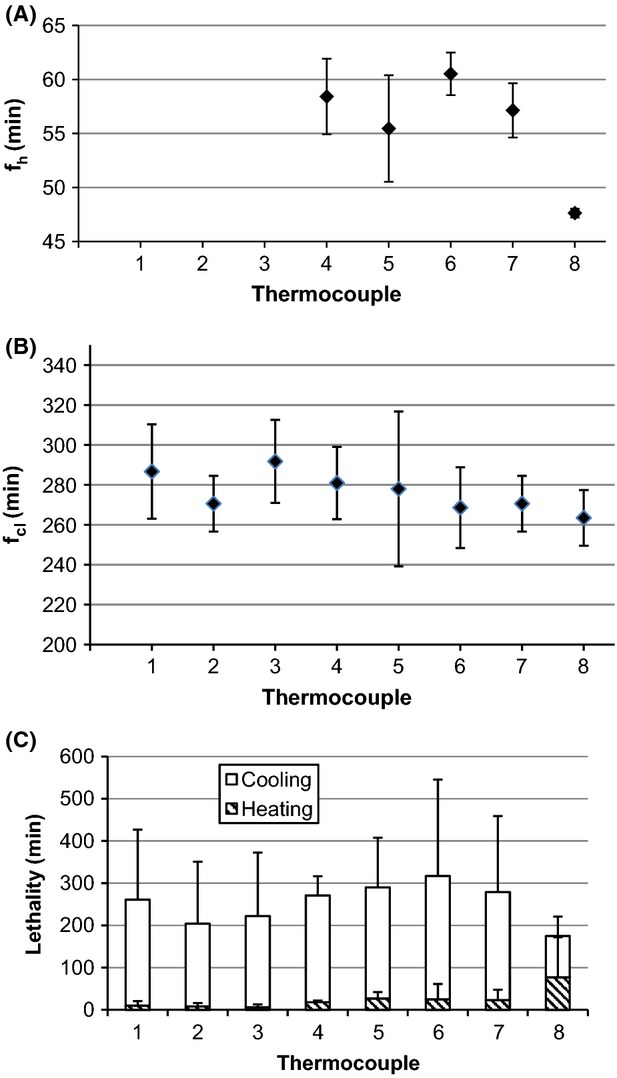
Average (95% CI) for (A) *f*_h_, (B) *f*_cl_, and (C) *F*_82.2°C_ for heating and cooling of applesauce, *n* = 3. Thermocouple location (height from bottom of container): 7.5 (geometric center), 6.5, 5.5, 5.0 (1/3 from the bottom), 4.5, 3.5, 2.5, and 1.5 cm, for thermocouple 1–8, respectively.

**Figure 2:**
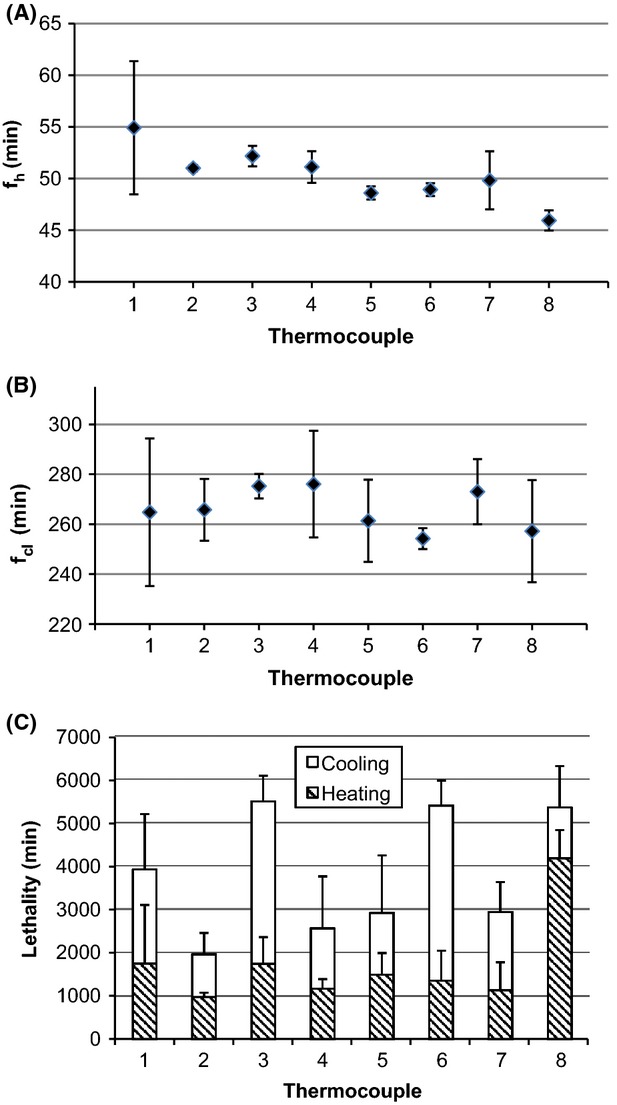
Average (95% CI) for (A) *f*_h_, (B) *f*_cl_, and (C) *F*_82.2°C_ for heating and cooling of tomato juice, *n* = 3. Thermocouple location (height from bottom of container): 7.5 (geometric center), 6.5, 5.5, 5.0 (1/3 from the bottom), 4.5, 3.5, 2.5, and 1.5 cm, for thermocouple 1–8, respectively.

**Figure 3:**
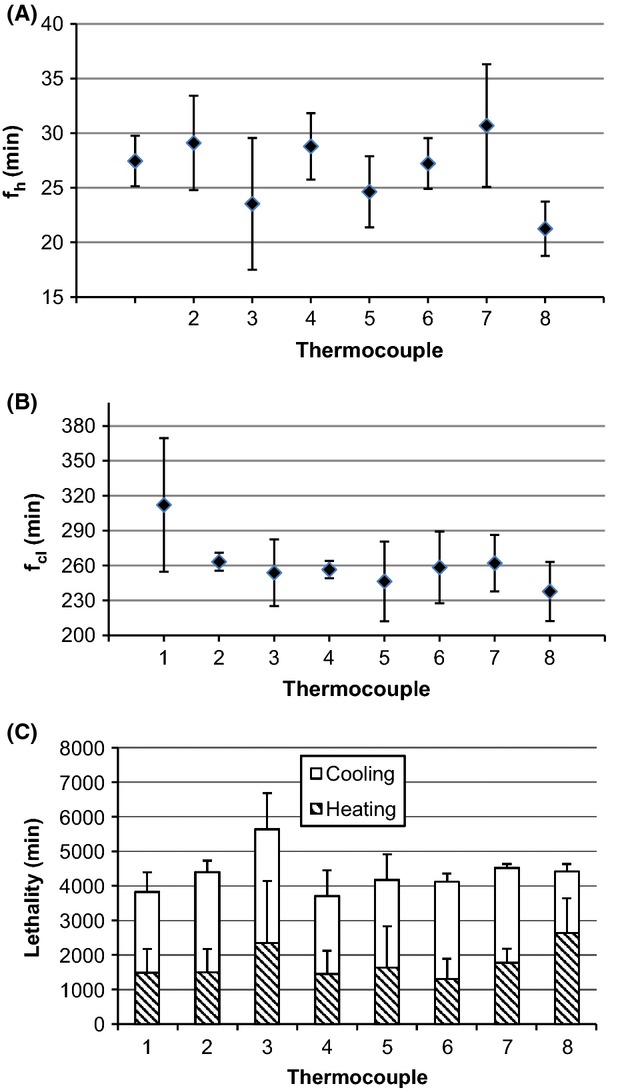
Average (95% CI) for (A) *f*_h_, (B) *f*_cl_, and (C) *F*_82.2°C_ for heating and cooling of cranberries in heavy syrup, *n* = 3. Thermocouple location (height from bottom of container): 7.5 (geometric center), 6.5, 5.5, 5.0 (1/3 from the bottom), 4.5, 3.5, 2.5, and 1.5 cm, for thermocouple 1–8, respectively.

Temperature versus time was plotted versus thermocouple location for home canning of applesauce, tomato juice, and cranberries in heavy syrup processed in a BWC. Rate of heating (*f*_h_) and cooling (*f*_cl_) and lethality (*F*_82.2°C_), overall and calculated for heating and cooling, were determined for each thermocouple location (Figs.1–3). The values of *f*_h_ and *f*_cl_ are a measure of the rate at which the temperature changes during heating and cooling, respectively; slower rates mean higher values for *f*_h_ and *f*_cl_. The time for a one-log reduction in the dimensionless temperature (*u*) for heating (*f*_h_) and cooling (*f*_cl_) are plotted in Figures3A and B). Products were packed hot into hot jars, processed in a BWC (99±1°C), and removed to the countertop for air cooling to 54°C. Processing times were 15, 35, and 15 min for applesauce, tomato juice, and cranberries in heavy syrup, respectively. It took, on average, 75 min, 175 min, and 150 min for applesauce, tomato juice, and cranberries, respectively, to cool to the target temperature of 54°C, *n* = 24.

The average value for *f*_h_ was 55 min for applesauce, 50 min for tomato juice, and 27 min for cranberries (*n* = 21) (Figs.3). For applesauce only, the temperature data did not enter the linear portion of the heating curve (*u* < 0.7) within the 15 min heating time at thermocouple locations 1–3, preventing calculation of *f*_h_ at those locations. From a statistical viewpoint, the value for *f*_h_ was weakly dependent on thermocouple location for tomato juice and cranberries, and strongly dependent on thermocouple location for applesauce (Table1). However, as pointed out later, these statistical differences in *f*_h_ were not physically meaningful. The value of *f*_cl_ for all foods did not statistically depend on thermocouple location (*P* > 0.05, *n* = 24) (Table1).

**Table 1:** Statistical analysis of data when processing applesauce, tomato juice, and cranberries in heavy syrup in pint jars, *n* = 3 trials per product.

	*P* value	
Main effect	Trial	Thermocouple
Applesauce		
Total *F*_82°C_	0.34	0.94
Heating *F*_82°C_	0.42	0.21
Cooling *F*_82°C_	0.43	0.72
*f*_h_	0.59	0.001*
*j*_h_	<0.0001*	0.25
*f*_cl_	0.1	0.51
*j*_cl_	0.26	0.001*
Tomato juice		
Total *F*_82.2°C_	0.96	0.13
Heating *F*_82.2°C_	0.48	0.0004*
Cooling *F*_82.2°C_	0.54	0.08
*f*_h_	0.88	0.02*
*j*_h_	0.01*	0.08
*f*_cl_	0.33	0.54
*j*_cl_	0.66	0.37
Cranberries in heavy syrup		
Total *F*_82.2°C_	0.01*	0.38
Heating *F*_82.2°C_	0.02*	0.28
Cooling *F*_82.2°C_	0.01*	0.01*
*f*_h_	0.04*	0.02*
*j*_h_	0.01*	0.08
*f*_cl_	0.08	0.07
*j*_cl_	0.002*	0.004*

* Indicates a significant difference (*P* < 0.05).

The values for *f*_h_ reported in the present work: applesauce *f*_h_ = 55 min and tomato juice *f*_h_ = 50 min, agree well with values reported by Ramakrishnan et al. (1987): applesauce *f*_h_ = 62 min and tomato juice *f*_h_ = 50 min. They also used pint jars in a BWC, but they did not report values of *f*_cl_ for cooling, did not use cranberries, and did not measure *f*_h_ and *f*_cl_ versus thermocouple location as we did in our study.

As shown by Ball and Olson (1957), and Kopelman and Pflug (1967), the values of *f*_h_ and *f*_cl_ depend on the Biot number *N*_Bi_ = *hd*_c_/*k*, where *h* is the convection heat transfer coefficient in the heat transfer medium, *k* is the thermal conductivity in the jar, and *d*_c_ is the jar radius. For pint jars, *d*_c_ = 4.1 cm. For boiling water, *h* = 3000–100,000 W/(m^2^ °K), and for air in free convection *h* ≈ 10 W/(m^2^ °K). For most foods, *k* ≈ 0.5 W/(m °K). For example, *k* for applesauce = 0.52 W/(m °K), *k* for tomato juice = 0.56 W/(m °K), and *k* for cranberries in heavy syrup = 0.50 W/(m °K), according to equation 4.7 in Singh and Heldman (2009). Therefore, *N*_Bi_ > 250 for heating the jar in a BWC, and *N*_Bi_ ≈ 0.8 for cooling the jar in air. As explained by Kopelman and Pflug (1967), when *N*_Bi_ > 50, conduction heat transfer inside the jar dominates, and convection heat transfer outside the jar does not limit the heating rate. Conversely, when *N*_Bi_ < 0.2, convection heat transfer outside the jar dominates, and conduction heat transfer inside the jar does not limit the cooling rate. This means that heating the jars in the BWC is fast and controlled by conduction inside the jar, and convection heat transfer in the kitchen air outside of the jar is slow and mostly, but not entirely, controls the cooling rate. In commercial canning operations, heated containers are partially, or completely, cooled in water at the end of the thermal process in order to ensure seal integrity, to maintain product quality, and to prevent spoilage by thermotolerant microorganisms (Weddig 2007). Therefore, in commercial canning operations, heating and cooling are assumed to occur at the same rate (*f*_h_ = *f*_cl_) because both heating and cooling occur with steam/water as the heat transfer medium. In contrast, in home canning, cooling takes place in air where *f*_h_ ≪ *f*_cl_.

The theoretical values of *f*_h_ and *f*_cl_ can be calculated and compared to the experimentally observed values. Using an infinite cylinder as a first approximation of a pint jar, *f*_h_*α*/(*d*_c_)^2^ = 0.40, where *α* is the thermal diffusivity, which is about 1.4±0.4 × 10^−7^ m^2^/s for most foods. The result is *f*_h_ = 60–110 min. This calculated value is similar to the experimentally observed value for applesauce (*f*_h_ = 55 min) and tomato juice (*f*_h_ = 50 min), but longer than observed for cranberries (*f*_h_ = 27 min). It is likely that some convection heating occurred inside of the jar when canning cranberries, speeding heat transfer, whereas the viscosity of applesauce and tomato juice impeded heat transfer and limited convection heating inside of the jar. Tomato juice is commonly considered to be a liquid, but without shear it is a weak gel (Augusto et al. 2013). Therefore, the tomato juice sits still in the jar like a gel, and heats by conduction just like the applesauce.

For cooling, the theoretical value is *f*_cl_*α*/(*d*_c_)^2^ = ½ln(10)/*N*_Bi_ or *f*_cl_ = 290 min. This calculated value agrees well with the observed value of *f*_cl_ = 270 min and confirms that convection heat transfer in the air controlled the cooling rate.

As pointed out by Ball and Olson (1957) and Kopelman and Pflug (1967), according to heat transfer theory the values of *f*_h_ and *f*_cl_ do not depend on thermocouple location. The values of *f*_h_ and *f*_cl_ depend only on *N*_Bi_, *α*, and *d*_c_. We confirmed this lack of dependence in our study, finding no dependence of *f*_cl_ on thermocouple location for any of the foods that we analyzed. There was a weak statistical difference in *f*_h_ with thermocouple location for tomato juice and cranberries (*P* = 0.02) and a statistically significant difference in *f*_h_ with thermocouple location for applesauce (*P* < 0.01). However, finding a statistically significant difference for *f*_h_ does not mean the difference is large. The actual differences in *f*_h_ with thermocouple location were small; coefficients of variation were 8% for applesauce, 5% for tomato juice, and 12% for cranberries. Overall, the results from these experiments confirm accepted theories of heat transfer: *f*_h_ and *f*_cl_ do not depend on thermocouple location.

We calculated lethality (*F*_82.2°C_) for the heating and cooling portions of the processing curves and integrated across the entire process (Figs.3 [C]). The come-up portion of the curve was disregarded in this study as past research has shown that the overall lethality contributed by the come-up process is minimal (Succar and Kayakawa 1982). There were no significant differences in *F*_82.2°C_ for the heating portion of the lethality curve versus thermocouple location for applesauce or cranberries (Table1). For tomato juice, only one of eight thermocouples (#8 located 1.5 cm from the base of the jar) had significantly more lethality during the heating portion of the lethality curve. Generally, *F*_82.2°C_ for the heating portion of the lethality curve did not depend on thermocouple location.

For the cooling portion of the lethality curve, *F*_82.2°C_ did not depend on thermocouple location for applesauce and tomato juice (Table1). Thermocouple placement was a significant factor only for cranberries, where *F*_82.2°C_ nevertheless fell in a narrow range; coefficient of variation was 18% CV. Therefore, there was only a weak dependence of *F*_82.2°C_ on thermocouple location for the cooling portion of the lethality curve for cranberries, and no dependence for applesauce and tomato juice.

When lethality was integrated across the heating and cooling portions of the curves, the total value of *F*_82.2°C_ was not statistically different (*P* > 0.05) versus thermocouple location for applesauce, tomato juice, and cranberries (Figs.3[C]).

In all cases, the majority of the overall lethality came from cooling: 90% for applesauce, and 60% for tomato juice and cranberries (*n* = 24). Thus, differences in the values of *f*_h_ with thermocouple location that were seen to be weakly associated for tomato juice and cranberries and strongly dependent for applesauce, did not matter in the final determination of *F*_82.2°C_. Because the heating portion contributed only 10% to the total value of *F*_82.2°C_ for applesauce, the differences in the value of *f*_h_ with thermocouple location did not matter in the final determination of *F*_82.2°C_. In conclusion, in each of the home-canning processes that we studied, the cooling portion, not the heating portion, was the major contributor to process lethality, and thermocouple location did not matter in the determination of overall calculated lethality.

Does the fact that *f*_h_ and *f*_cl_ do not depend on thermocouple location mean there is no “cold spot” in the jar during a home-canning operation? Yes and no. During heating, the geometric center is the last to heat, and remains coldest the longest. But during cooling it is also the last to cool. The cold spot during heating becomes the hot spot during cooling. The heating and cooling rates (*f*_h_ and *f*_cl_) do not depend on thermocouple location. There is simply a lag time that shifts the heating and cooling periods in time; a longer time shift as the thermocouple is placed further away from the heating medium. For example, at the geometric center, the lag time is the longest, and the temperature continues to increase for a short time period after heating stops and cooling begins. The net impact of thermocouple placement on the calculated integrated lethality, or the area under the curve of the lethal rate versus time, is negligible in home canning of acid foods where established recipes instruct consumers to place jars of hot food on the counter to air cool after processing in a BWC.

Ensuring food safety is a major concern for thermal processing operations. For high-acid foods, a 5-log reduction in the target pathogen generally provides a sufficient margin of safety. The log reduction value (LRV) is given by: LRV = *F*_82.2°C_/*D*_82.2°C_. The average values of *F*_82.2°C_ calculated in our study were: applesauce = 250 min, tomato juice = 3900 min, and cranberries = 4200 min (*n* = 72). Peng et al. (2012) calculated a *D*_82.2°C_ = 78 min for spores of *Bacillus coagulans* 185A in tomato juice at pH 4.3. Mazzotta (2001) studied the heat tolerance of *E. coli* O157:H7 in fruit juices and determined a *D*_82.2°C_ = 0.00008 min for vegetative cells. Our results clearly demonstrate the inherent safety of home-canning practices for high-acid foods when a tested recipe is followed.

## Conclusions

We determined that thermocouple location in a jar during home canning did not affect the calculated lethality, in agreement with heat transfer theory that predicts that the rate of heating and cooling (*f*_h_ and *f*_cl_) do not depend on thermocouple location. Experimentally, there were some minimal differences in the rate of heating, but no differences in the rate of cooling versus thermocouple location in the jar. We also found that there were some minimal differences in lethality during heating, but no differences in lethality during cooling versus thermocouple location. Because most of the lethality attained in a home-canning process occurs during the cooling portion, the minor differences in the heating portion of the process do not affect the overall lethality of the process. There was no significant difference in lethality versus thermocouple location when integrated across both the heating and cooling portions of the curve. Thus, as long as up-to-date, research-tested home-canning practices are followed for high-acid foods, consumers can be reassured by the inherent safety of the product that they are producing for themselves and their families.

## Supporting Information

**Table S1.** Mean lethality as determined by thermocouple location when processing applesauce, tomato juice, and cranberries in heavy syrup in pint jars.
