# Supplementary material for: Heat penetration and thermocouple location in home canning
Source: Food Sci Nutr. 2014 Dec 9;3(1):25–31. doi: 10.1002/fsn3.185 (PMC4304559; doi:10.1002/fsn3.185)
Supplement: Supplementary file 1 [file fsn30003-0025-sd1.docx]

**Supplementary File Contents**

**Table 1. Mean lethality as determined by thermocouple location when processing applesauce, tomato juice, and cranberries in heavy syrup in pint jars.**

| Food | TC^a^ | f_h_ (min) | j_h_ | f_cl_ (min) | j_cl_ | F_82°C_^b^ (min) | F_82°C_^c^ (min) | F_82°C_^d^ (min) |
| --- | --- | --- | --- | --- | --- | --- | --- | --- |
| Applesauce | 1 | n/a^e^ | n/a | 287 | 1.34 AB^f^ | 10 | 251 | 261 |
|  | 2 | n/a | n/a | 271 | 1.39 AB | 8 | 196 | 203 |
|  | 3 | n/a | n/a | 292 | 1.44 A | 6 | 216 | 222 |
|  | 4 | 58 A | 1.88 | 281 | 1.19 C | 18 | 253 | 272 |
|  | 5 | 56 A | 1.94 | 278 | 1.19 CD | 27 | 263 | 290 |
|  | 6 | 61 A | 1.99 | 269 | 1.34 AB | 25 | 292 | 317 |
|  | 7 | 57 A | 1.89 | 271 | 1.24 BC | 23 | 256 | 278 |
|  | 8 | 48 B | 1.91 | 264 | 1.04 D | 77 | 98 | 175 |
| Tomato  Juice | 1 | 55 A | 1.97 | 265 | 1.19 | 1749 B | 1856 | 3605 |
|  | 2 | 51 AB | 2.60 | 266 | 1.18 | 977 B | 1300 | 2276 |
|  | 3 | 52 AB | 2.27 | 275 | 1.20 | 1741 B | 3241 | 4983 |
|  | 4 | 51 AB | 2.33 | 276 | 1.13 | 1169 B | 1814 | 2983 |
|  | 5 | 49 BC | 2.35 | 261 | 1.42 | 1491 B | 2236 | 3727 |
|  | 6 | 50 BC | 2.35 | 254 | 1.19 | 1358 B | 3460 | 4818 |
|  | 7 | 50 BC | 2.25 | 273 | 1.15 | 1135 B | 2109 | 3243 |
|  | 8 | 46 C | 2.00 | 257 | 0.94 | 4178 A | 1633 | 5811 |
| Cranberries | 1 | 28 ABC | 3.14 | 312 | 1.03 ABC | 1495 | 2336 BC | 3761 |
|  | 2 | 29 AB | 2.92 | 263 | 1.05 AB | 1502 | 2895 AB | 4328 |
|  | 3 | 24 CD | 3.94 | 254 | 1.09 A | 2351 | 3290 A | 5507 |
|  | 4 | 29 AB | 2.74 | 257 | 1.02 ABC | 1458 | 2252 BC | 3618 |
|  | 5 | 25 BCD | 3.20 | 246 | 0.95 DC | 1650 | 2525 B | 4076 |
|  | 6 | 27 ABC | 3.13 | 258 | 1.04 ABC | 1311 | 2811 AB | 3948 |
|  | 7 | 31 A | 2.56 | 262 | 0.96 BCD | 1782 | 2742 AB | 4324 |
|  | 8 | 21 D | 3.90 | 238 | 0.86 D | 2645 | 1775 C | 4309 |

^a^TC = Thermocouple location in each pint jar (from base) – numbers 1-8 corresponding to height from the base equivalent to 7.5, 6.5, 5.5, 5.0, 4.5, 3.5, 2.5,and 1.5 cm, respectively.

^b^ Lethality corresponding to heating portion of temperature curve, excluding come up time. With T_R_ = 82.2 °C (180 °F), z = 5.5 °C, and D_82°C_ = 0.005 min with *Escherichia coli* O157:H7 as the target organism.

^c^ Lethality corresponding to cooling portion of temperature curve. With T_R_ = 82.2 °C (180 °F), z = 5.5 °C, and D = 0.005 min with *Escherichia coli* O157:H7 as the target organism.

^d^ Integrated lethality for the entire process, excluding come up time. With T_ref_ = 82.2 °C (180°F), z=5.5 °C , and D_82°C_ = 0.005 min with *Escherichia coli* O157:H7 as the target organism.

^e^Data did not enter the linear portion of the heating curve (u < 0.7) at these TC locations preventing calculation of f_h_ and j_h_.

^f^Means followed by the same letter (A-D) for a given food product in a column are not significantly different (p > 0.05), (n = 3). Protected LSD test only performed for variables that proved to be significantly different with ANOVA.
